# Glucagon-like peptide-1 agonists in children with obesity and type 2 diabetes. an umbrella review

**DOI:** 10.3389/fendo.2026.1777907

**Published:** 2026-02-19

**Authors:** Hyder Mirghani, Laila Albishi, Sawsan Mohmmad Alblewi

**Affiliations:** 1 Internal Medicine Department, Faculty of Medicine, University of Tabuk, Tabuk, Saudi Arabia; 2 Pediatrics Department, Faculty of Medicine, University of Tabuk, Tabuk, Saudi Arabia

**Keywords:** adolescents, blood pressure, children, GLP-1 agonists, HbA1c, hypoglycemia, obesity, side effects

## Abstract

**Introduction:**

Obesity and type 2 diabetes mellitus (Type 2 DM) are rising at an alarming rate among children and adolescents. This population often exhibits suboptimal glycemic control and diabetes-related complications. Glucagon-like peptide-1 receptor agonists (GLP-1 agonists) have emerged as a promising therapeutic option for pediatric patients due to their beneficial effects on weight reduction and glycemic regulation. Literature on this important issue is scarce. We aimed to assess the effects of GLP-1 agonists on body weight, HbA1c, body mass index z (BMI z), and systolic blood pressure (SBP). Additionally, we discussed adverse events and hypoglycemia.

**Methods:**

We searched PubMed/MEDLINE, Web of Science, and the Cochrane Library from October to November 2025 using the following terms: GLP-1 agonists, semaglutide, tirzepatide, liraglutide, exenatide, children, obesity, adolescents, blood pressure, BMI z, hypoglycemia, body weight, and HbA1c. We retrieved 1600 articles. Out of the 44 reviews found, only 11 meta-analyses were included in the final results.

**Results:**

GLP-1 agonists were more effective than control in reducing body weight, HbA1c, BMI z, and blood pressure, MD = 0.11, 95% CI 0.05–0.25, MD = 0.65, 95% CI 0.53–0.80, MD = 0.85, 95% CI 0.81–0.90, and MD = 0.19, 95% CI 0.04–83, respectively. The total adverse events and hypoglycemia were not different, log ratios=1.29, 95% CI 0.80–2.09, and log ratios=1.26, 95% CI 0.59–2.70, respectively.

**Discussion:**

GLP-1 agonists are a promising and effective therapy for lowering weight, HbA1c, BMI z, and SBP in adolescents with obesity or youth with type 2 DM. Moreover, GLP-1 agonists were well tolerated, and the total adverse events and hypoglycemia were comparable to those of controls.

## Introduction

Diabetes and obesity are major health concerns, with diabetes mellitus affecting 10.5% globally. The prevalence is expected to increase in 2045 by 21.1% in middle-income countries, and 12.1% in high-income countries (1). Recent data from the International Diabetes Federation indicate that the Middle East and North Africa region (MENA) currently bears the world’s highest age-adjusted prevalence of diabetes among adults at 12.2%. This burden is expected to rise to 13.3% by 2030, and projections suggest that by 2045, approximately one in eight individuals in the region will be living with diabetes (2). In some countries in this diabetes super-region, type 2 DM affected more than a quarter of the adult population in Saudi Arabia, with an overall prevalence of about 28% according to 2016 and 2022 data. Adults older than 40 years showed a substantially higher likelihood of having T2DM, with nearly double the risk compared with those under 40 years of age, indicating age as a strong determinant of disease burden in the general population (3). Obesity affects 20% of children and adolescents, with a parallel increase in type 2 DM in the young age group (2). A systematic review and meta-analysis reported that the pooled prevalence of obesity among children and adolescents was 8.5% (95% CI 8.2–8.8), highlighting a substantial public health burden. Despite the ongoing rise in diabetes and obesity rates. Nonetheless, significant challenges hinder the translation of existing evidence into routine clinical practice. These barriers include poor integration of obesity and diabetes services across different levels of care within healthcare systems, inadequate training of healthcare professionals, and limited affordability and accessibility of prevention and treatment services (4). The increasing rate of type 2 DM in children and adolescents is mainly due to the increasing obesity from an unhealthy diet and lack of physical activity in genetically predisposed people (5–7). The number of newly diagnosed type 2 diabetes cases in children and adolescents was 41,600 in 2017 worldwide (8). Genome-wide association studies have revealed multiple genetic regions linked to the risk of type 2 DM, with variants in transcription factor 7-like 2, peroxisome proliferator-activated receptor gamma, and fat mass and obesity–associated genes influencing insulin secretion and sensitivity, thereby highlighting the genetic basis of disease susceptibility (9). Children and adolescents with type 2 DM have poor glycemic control and are more prone to diabetes microvascular complications (10). In addition, type 2 DM in the pediatric age group is associated with several cardiovascular risk factors, including hypertension, dyslipidemia, and metabolic-associated liver disease (11). Because of the above, the use of GLP-1 agonists is on the rise worldwide.

GLP-1 agonists use among patients with type 2 DM started with the approval of the twice/daily exenatide in 2005. Many classes were developed in the following years. Liraglutide injection once daily and semaglutide once/week subcutaneous injection were approved in 2017 for glycemic control, and in 2021 for the treatment of obesity (12–14). Liraglutide is currently approved for use in pediatrics, with some concerns regarding treatment adherence. Although semaglutide was approved and showed better glycemic and weight control. However, the recruitment of children for controlled trials is challenging (15–17). Other GLP-1 agonists that received approval for use in the pediatric age group are exanetide slow-release and dulaglutide. GLP-1 agonists were shown to reduce glycemic parameters, reduce weight, and improve obesity associated comorbidities (18). The use of GLP-1 agonists was shown to reduce weight and improve glycemic control in children through appetite reduction and slowing gastric emptying. GLP-1 agonists act on the brain by stimulating specific receptors in central nervous system regions such as the hypothalamus, leading to enhanced feelings of fullness. They also influence gastrointestinal function by slowing gastric emptying, which helps blunt post-meal rises in lipids and carbohydrates. This gastric effect is partly regulated through neural signaling pathways involving the vagal afferent nerves, brainstem nuclei such as the nucleus of the solitary tract, and vagal efferent fibers (19). In addition, they increase insulin secretion and decrease glucagon secretion. In large single trials, such as *SCALE Teens* (liraglutide) and *STEP TEENS* (semaglutide), reductions of 4–16% in BMI were observed over 56–68 weeks. However, because of ongoing growth and pubertal development, extrapolation from adult data must be cautious (20–22). The findings from earlier meta-analyses, demonstrated a consistent reduction in body weight. However, evidence related to glycemic control remains inconsistent across studies, and several meta-analyses did not evaluate key clinical outcomes such as body mass index z score, systolic blood pressure, overall adverse events, and hypoglycemia, which are critical in managing pediatric obesity and diabetes. Consequently, synthesizing all available evidence through a comprehensive umbrella review is highly warranted. This umbrella review aimed to assess the role of GLP-1 agonists on body weight, HbA1c, waist circumference, BMI z, and systolic blood pressure in children and adolescents.

## Methods

This umbrella review was conducted according to the PRISMA guidelines with additional methodological considerations specific to umbrella reviews. This umbrella review aimed to study the effects of GLP-1 agonists on pediatric body weight, HbA1c, BMI z, SBP, gastrointestinal adverse events, and hypoglycemia in children with obesity/type 2 DM.

### Inclusion criteria

We included systematic reviews and meta-analyses assessing the role of GLP-1 agonists on weight, HbA1c, BMI z, SBP, gastrointestinal adverse events, and hypoglycemia in children with obesity/type 2 DM. The studies must report the mean difference, standard mean difference, odds ratios, hazard ratios, and risk ratios or equivalent with corresponding 95% confidence intervals for the outcomes.

### Exclusion criteria

Observational studies, trials, cross-sectional studies, case-control studies, opinion, editorials, letters to the editors, and narrative reviews were not included. In addition, studies not reporting the mean difference, standard mean difference, odds ratios, hazard ratios, and risk ratios for the outcomes were not included.

### Outcome measures

The outcomes measures were the effects of GLP-1 agonists on pediatric body weight, HbA1c, BMI z, SBP, gastrointestinal adverse events, and hypoglycemia in children with obesity/type 2 DM.

### Literature search

**Figure 1:**
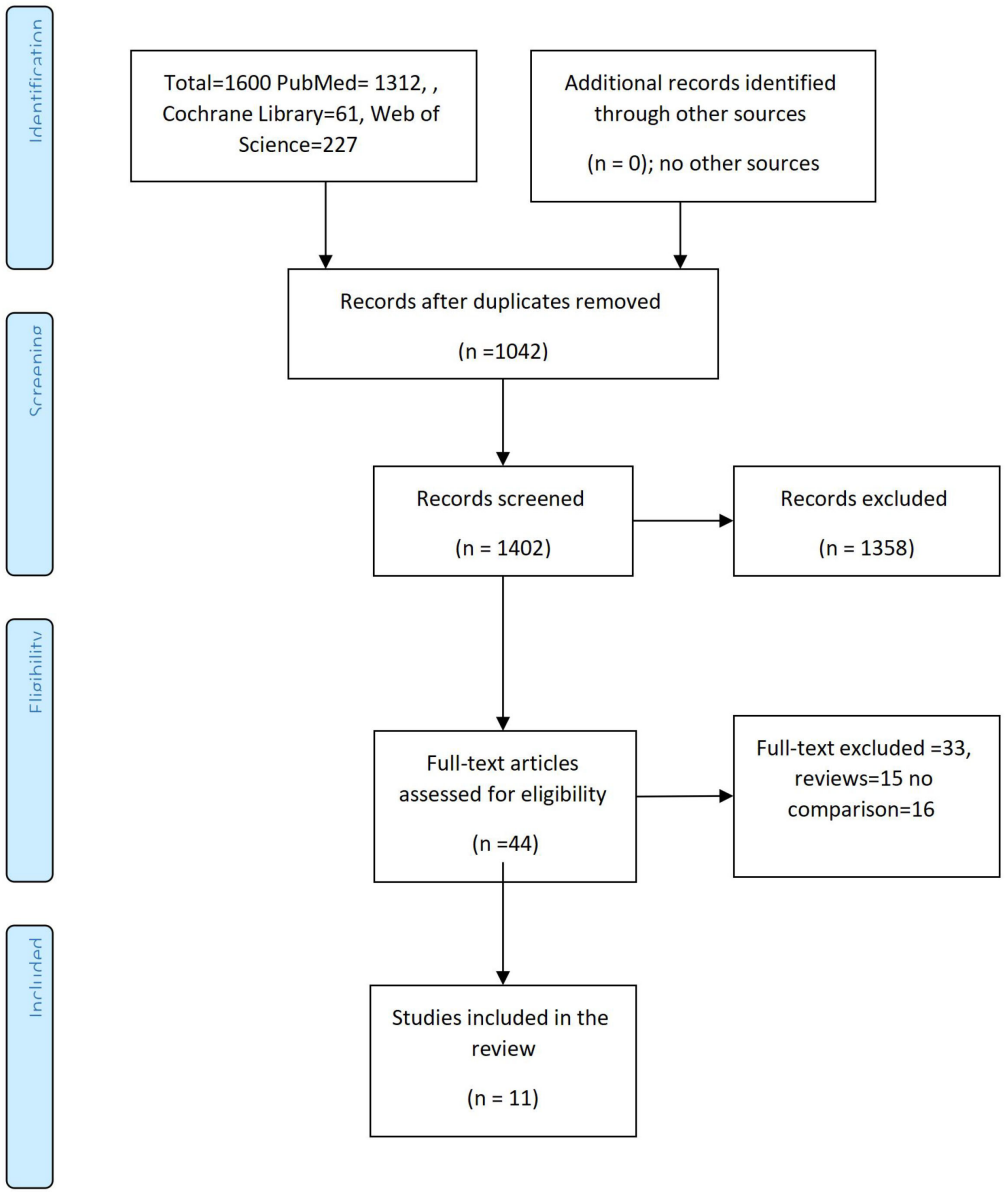
Studies evaluating the role of GLP-1 agonists in children with obesity/type 2 diabetes. The PRISMA Chart.

We reviewed the literature in PubMed/MEDLINE, Web of Science, and Cochrane Library from inception up to December 2025 using the following terms: GLP-1 agonists, semaglutide, tirzepatide, liraglutide, exenatide, children, obesity, adolescents, BMI z, blood pressure, gastrointestinal, adverse events, hypoglycemia, and HbA1c. We retrieved 1600 articles (1312 articles in PubMed, 227 in the Web of Science, and 61 in Cochrane Library), of which 1402 remained after the removal of duplications, and 44 reviews and meta-analyses full texts were reviewed. Out of the 44 reviews found, only 11 meta-analyses were included in the final results **Figure 1**.

### Data extraction

The data from 11 systematic reviews were reported in **Table 1**. The author’s name, year of publication, weight reduction, BMI z, SBP, HbA1c, gastrointestinal adverse events, and hypoglycemia were reported **Tables 1**, **2**.

**Table 1:** Systematic reviews and meta-analyses assessing the effects of GLP-1 agonists on obesity and type 2 diabetes mellitus in children and adolescents.

Author	HbA1c outcome	Adverse events outcome	Weight outcome	BMI, Z outcome	SBP outcome	Hypoglycemia outcome
Chadda et al., 2021 (23)	MD -0.30; 95% CI -0.57, -0.04	Not assessed	MD; -1.86; 95% CI -2.60, to -1.13	MD -0.12; 95% CI -0.22, -0.03	Not assessed	Not assessed
Cornejo-Estrada et al., 2023 (24)		RR 1.10; 95%CI, 0.64 to 1.90 total	MD; −2.62, 95%CI, −6.35 to 1.12	Not assessed	Not assessed	RR 1.08; 95%CI, 0.37 to 3.15
Dai et al., 2024 (25)	RD; -0.34, 95% CI, -0.51, to -0.18	Not assessed	RD; -4.28 95% CI, 6.95, -1.60	Not assessed	Not assessed	Not assessed
Gou et al., 2023 (26)	WMD: -0.29; 95% CI: -0.52, -0.06	Not assessed	WMD: -2.13; 95%CI: -4.23, to -0.03	Not assessed	Not assessed	Not assessed
Katole et al., 2024 (27)		OR; 3.06, 95%CI, 2.12, 4.42, GIT	MD -4.98, 95% CI, -8.49, -1.46	MD; -0.35, 95%CI -0.72, -0.01	Not assessed	Not assessed
Kotecha et al., 2025 (28)	MD-0.44; 95% CI, -0.68% to -0.21	RR; 0.73; 95% CI, 0.38 to 1.07	MD; -3.02; 95% CI, -4.98 to -1.06	Not assessed	MD; -2.73 mm Hg; 95% CI, -4.04 to -1.43	RR; 0.51; 95% CI, -0.07 to 1.08
Romariz et al., 2025 (29)		RR 1.52; 95% CI 1.09 to 2.12, GIT	MD -4.32; 95% CI -7.02 to -1.63	MD -0.28; 95% CI -0.45 to -0.1	Not assessed	Not assessed
Ryan et al., 2021 (30)	MD -0.24; 95% CI, -0.44,-0.05	RR;1.69, 95% CI, 0.95 to 3.10	MD -1.50; 95% CI, -2.50, to -0.50	MD -0.14, 95% CI, -0.23, to -0.06	MD -2.30, 95% CI, -4.11, to -0.49	Not assessed
Sedenho-Prado et al., 2025 (31)	Not assessed	Not assessed	SMD −0.60; 95% CI −0.89 to −0.44		SMD −0.20; 95% CI −0.35 to −0.04	Not assessed
Wang et al., 2024 (32)	0.37	RR; 0.67, 95% CI, 0.40 to 1.02	MD; -2.89; 95% CI, -5.12 to -0.65	MD; -0.22, 95% CI, -0.45 to 0.01	MD; -2.31, 95% CI, -2.96 to -1.65	Not assessed
Yugar et al., 2024 (33)	MD -1.01, 95% CI, -1.26; -76	Not assessed	MD -1.6, 95% CI, -2.83;-3.6	Not assessed	MD -0.19; 95% CI, -3.9;- 3.52	OR 2.03; 95% CI, 1.16 to 3.54

**Table 2:** Study characteristics and AMSTAR-2 quality assessment of meta-analyses on GLP-1 agonists in children and adolescents.

Study	Overall AMSTAR-2 confidence	Journal/year	Studies included	Participants age	Patients number	Morbidities
Chadda et al., 2021 (23)	Moderate	Obes Rev 2021	9 trials	< 18	286	DM/obesity
Cornejo-Estrada et al., 2023 (24)	Low	Children 2023	3 trials	5-< 18	296	Obesity
Dai et al., 2024 (25)	Low	J Clin Res Pediatr Endocrinol 2024	14 trials	< 18	1262	DM/obesity
Gou et al., 2023 (26)	Low–Moderate	Eur J Pediatr 2023	7 studies	Not stated	547	Obesity
Katole et al., 2024 (27)	Critically low	Cureus 2024	7 trials	< 18	567	Obesity
Kotecha et al., 2025 (28)	High	JAMA Pediatr 2025	18 trials	6 to17	1402	DM/obesity
Romariz et al., 2025 (29)	High	Pediatr Res 2025	11 trials	6 to 16	1024	Obesity
Ryan et al., 2021 (30)	Moderate	J Pediatr 2021	9 studies	Not stated	474	Obesity
Sedenho-Prado et al., 2025 (31)	High	Int J Obes 2025	8 studies	≤ 18	715	Obesity
Wang et al., 2024 (32)	High	Obes Rev 2024	15 trial	Not stated	1286 y	DM/obesity
Yugar et al., 2024 (33)	High	Diabetol Metab Syndr 2024	5 studies	10-18	415	Diabetes

### The quality assessment of the included meta-analyses

The quality of the included meta-analyses was assessed by the Measurement Tool to Assess Systematic Reviews, version 2 (34) **Table 3**.

**Table 3:** Analysis of the quality of evidence by grading of recommendations assessment, development, and evaluation.

Outcome	Studies	Study design	Risk of bias	Inconsistency	Indirectness	Imprecision	Other considerations	Certainty of evidence
Body weight	11	Trials=11	serious	Serious (*I^2^* = 76%)	Not serious	Not serious	None	⨁⨁◯◯ LOW
HbA1c	6	Trials=6	Serious	Not serious (*I^2^* = 81%)	Not serious	Not serious	None	⨁⨁◯◯ LOW
BMI z	5	Trials=3	serious	Very serious (*I^2^* = 1%)	Not serious	Not serious	None	⨁⨁◯◯ LOW
GIT adverse events	6	Trials=3	Serious	Not serious (*I^2^* = 85)	Not serious	Not serious	None	⨁⨁◯◯ LOW
Systolic blood pressure	5	Trials=3	serious	Serious (*I^2^* = 93%)	Not serious	Not serious	None	⨁⨁◯◯ LOW
Hypoglycemia	3	Trials=2	Serious	Not serious (*I^2^* = 48%)	Not serious	Not serious	None	⨁⨁◯◯ LOW

### Overlap determination

The Overlap of primary studies across meta-analyses was evaluated by identifying shared cohorts and calculating the Corrected Covered Area (CCA) (35). There were 11 meta-analyses with 78 trials included; the duplicates were 66, with only 12 unique trials. Therefore, the Corrected Covered Area (55% (very high overlap).

### Statistical analysis

We used the RevMan System from Cochrane (version 5.4, Oxford) for data analysis. The retrieved mean differences (MD) for weight, HbA1c, SBP, and BMI z, odds ratios, and hazard ratios for gastrointestinal side effects and hypoglycemia were converted to Log ratios and standard errors (SE). MD, Log ratios, and SE were entered using the random effect to generate the forest plots at 95% CI, and a standard error of 5%. A funnel plot was generated for the weight and HbA1c due to the significant heterogeneity observed. A heterogeneity of 25% was considered mild, and heterogeneity of ≥ 50% was considered significant. A subgroup analysis was conducted for the HbA1c outcome to locate the source of heterogeneity. A P-value of < 0.05 was considered significant.

## Results

**Figure 2:**
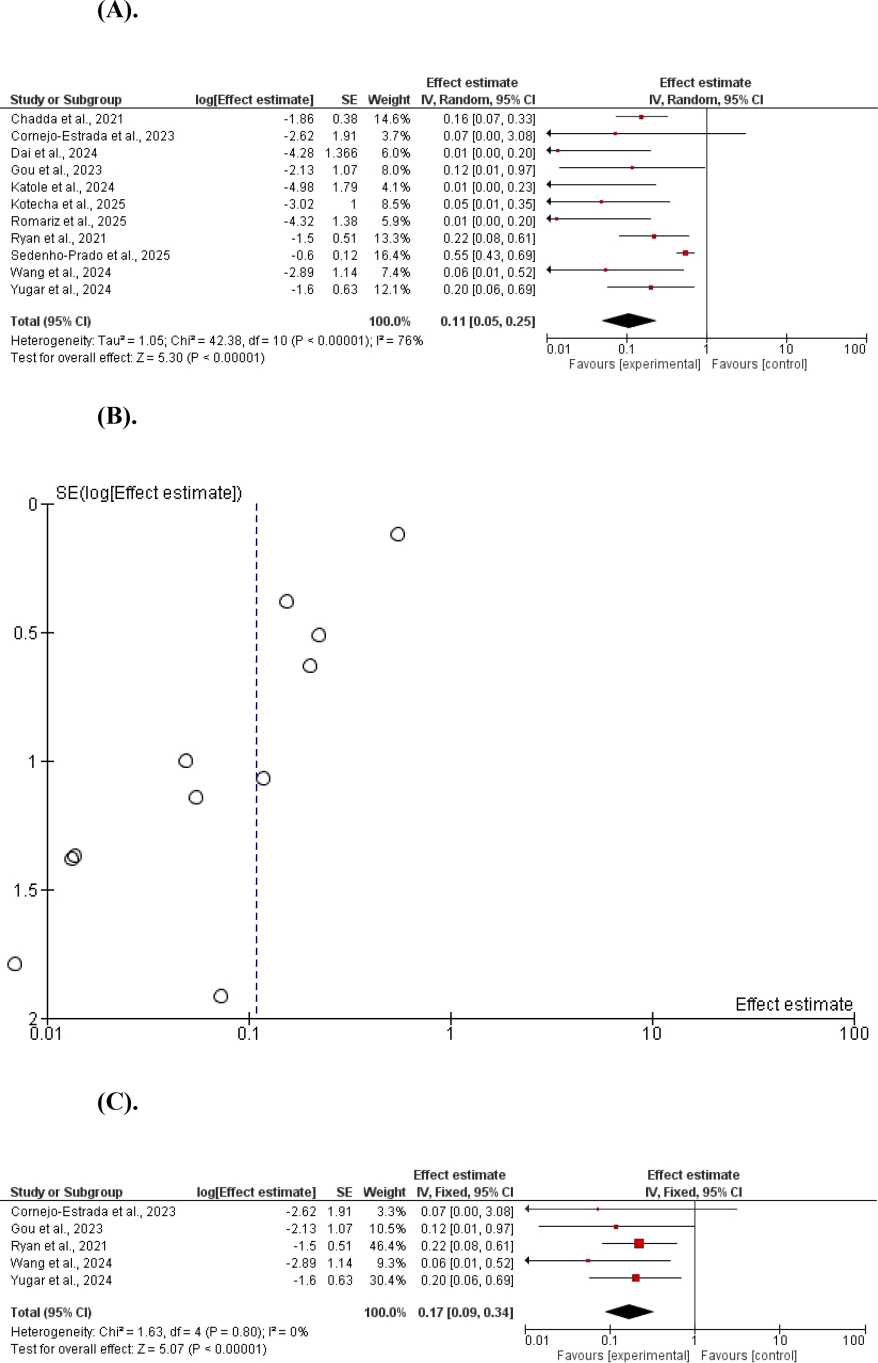
**(A)** The effect of GLP-1 agonists on body weight in children (forest plot). **(B)** The effect of GLP-1 agonists on body weight in children (funnel plot). **(C)** The effect of GLP-1 agonists on weight in children (forest plot, no heterogeneity).

A meta-analysis of eleven studies demonstrated a statistically significant reduction in body weight in the GLP-1 agonists compared with controls. Using a random-effect model, the pooled mean difference (MD)=0.11, 95% CI 0.05–0.25; Z = 5.30, P < 0.00001. There was evidence of between-study heterogeneity (χ^2^ = 42.38, df = 10, P < 0.001; I^2^ = 076%). A fixed-effects model produced identical results, indicating robust findings independent of the analytical approach. Weight reduction was significant after removing studies with a significant contribution to heterogeneity, MD = 0.17, 95% CI 0.09–0.34; Z = 5.07, P < 0.00001. There was no evidence of between-study heterogeneity (χ^2^ = 1.63, df = 4, P = 0.80; I^2^ = 0%) (**Figures 2A–C**).

**Figure 3:**
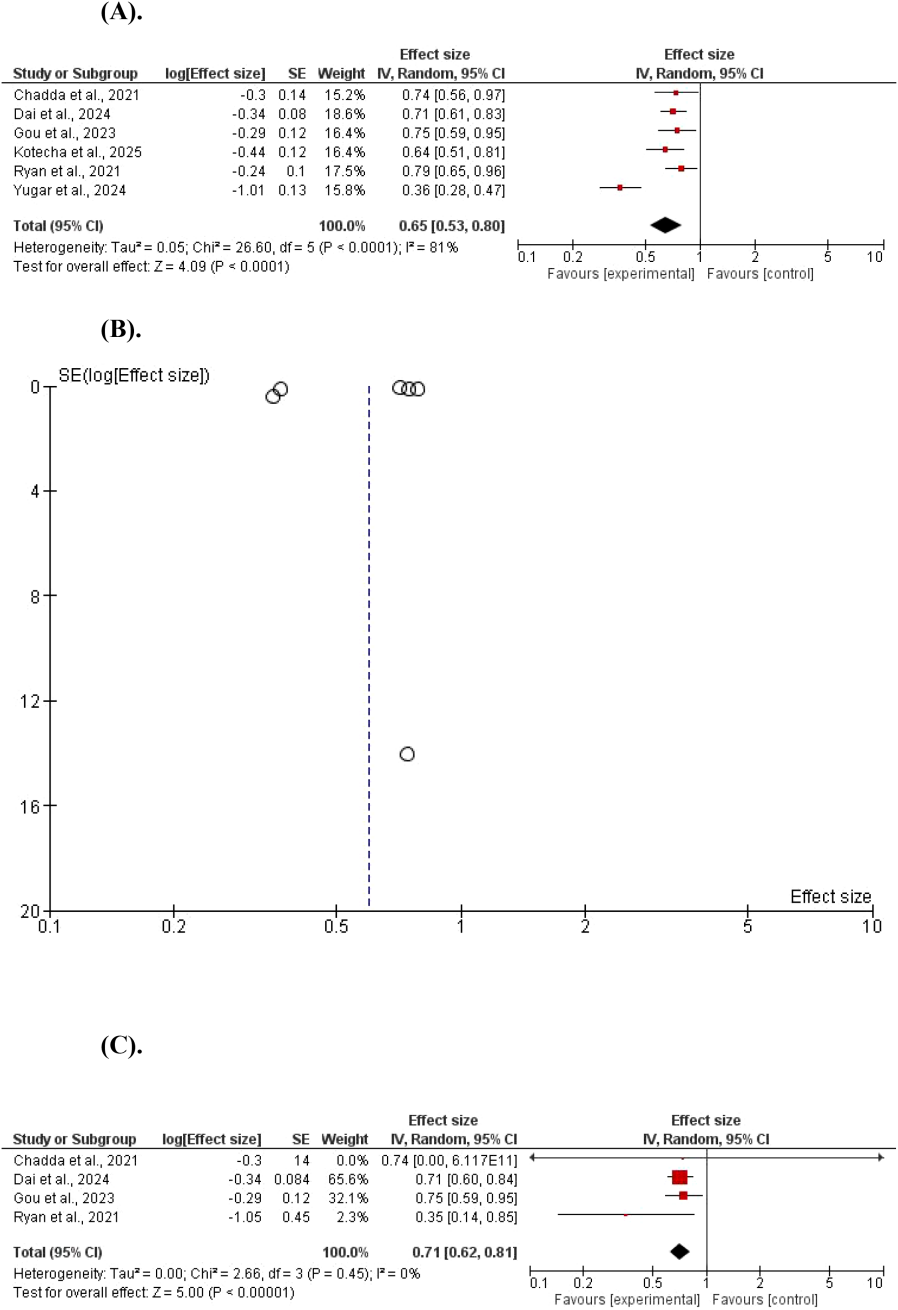
**(A)** The effect of GLP-1 agonists on HbA1c in children. **(B)** The effect of GLP-1 agonists on HbA1c in children (funnel plot). **(C)** The effect of GLP-1 agonists on HbA1c in children (Forest Plot, no heterogeneity).

Regarding the HbA1c, GLP-1 agonists reduced it with a significant statistical difference, MD = 0.65, 95% CI 0.53–0.80; P< 0.001, and Z = 4.09. A significant heterogeneity was found (χ^2^ = 26.60, df = 5, P < 0.001; I^2^ = 81%). A subgroup analysis showed a significant HbA1c reduction, MD = 0.71, 95% CI 0.62–0.81; P < 0.001, and Z = 5.00. No significant heterogeneity was found (χ^2^ = 2.66, df = 3, P = 0.45; I^2^ = 0%) (**Figures 3A–C**).

**Figure 4:**
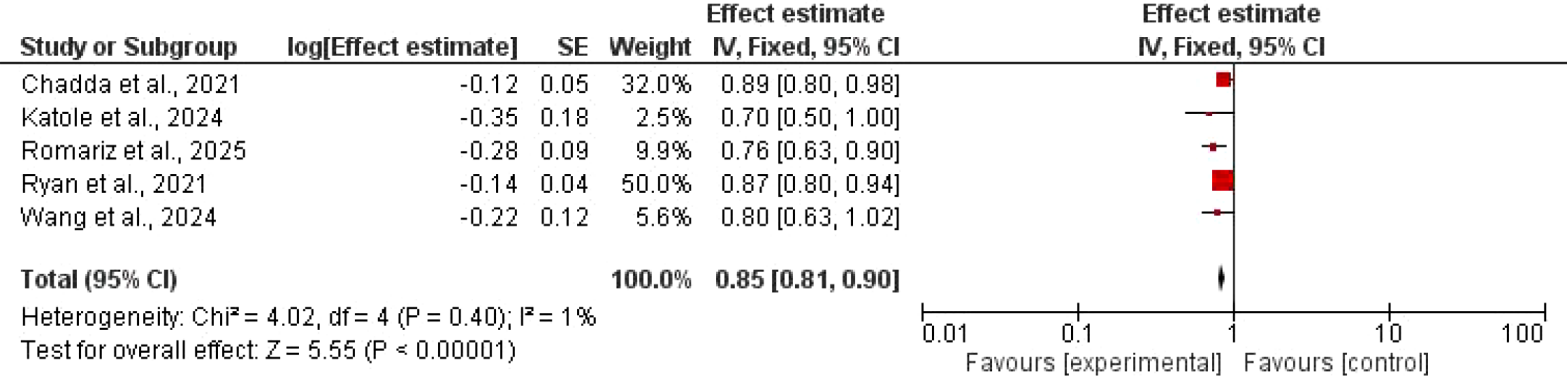
The effect of GLP-1 agonists on BMI z in children.

Only 6 studies assessed the BMI z; there was a significant statistical reduction in GLP-1 agonists compared to controls, MD = 0.85, 95% CI 0.81–0.90; P < 0.001, and Z = 5.55). No significant heterogeneity was found (χ^2^ = 4.02, df = 4, P = 0.40; I^2^ = 1%) **Figure 4**.

**Figure 5:**
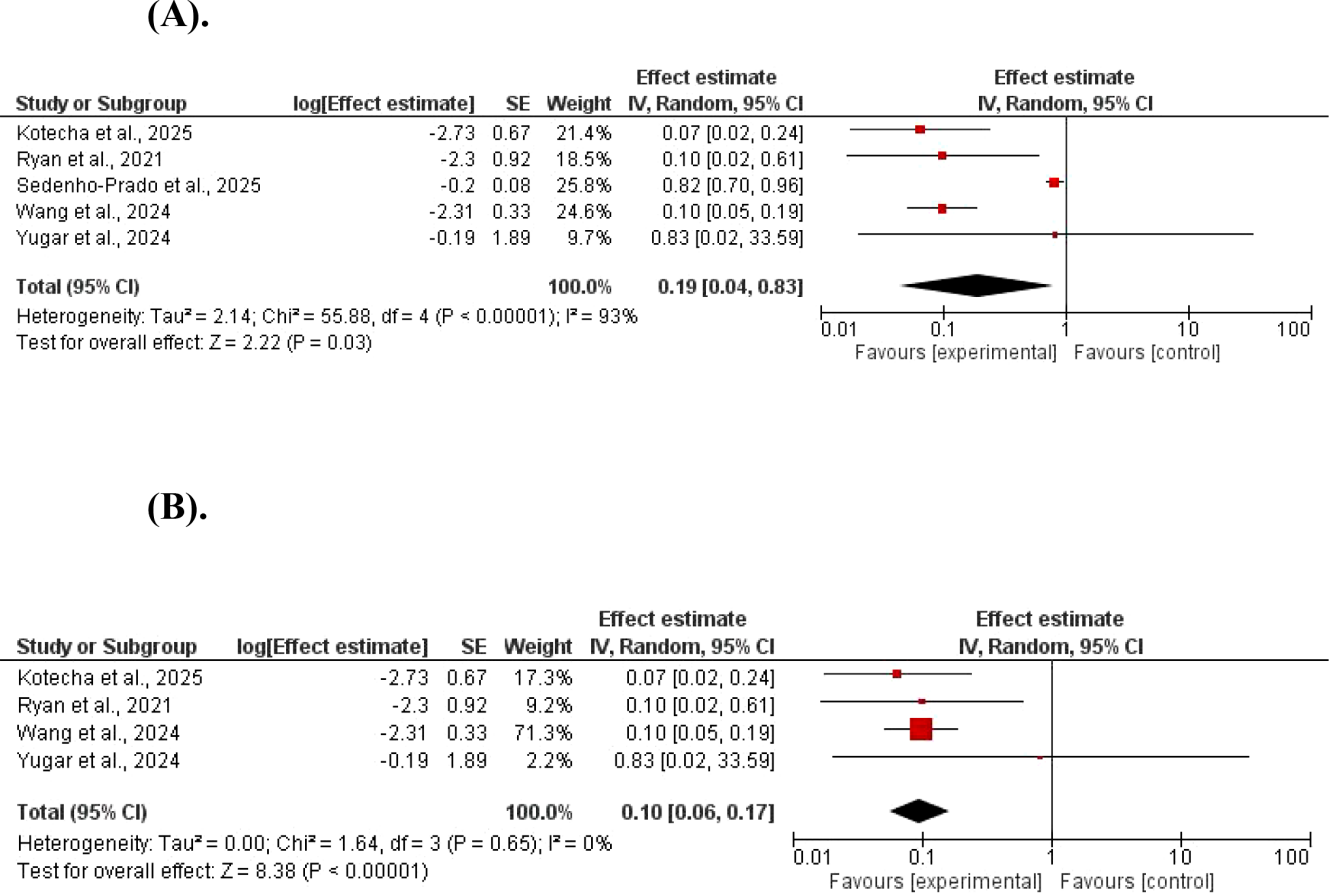
**(A)** The effect of GLP-1 agonists on systolic blood pressure in children. **(B)** The effect of GLP-1 agonists on systolic blood pressure in children (no heterogeneity).

The SBP was significantly reduced by GLP-1 agonists, MD = 0.19, 95% CI 0.04–83; P, 0.03, and Z = 2.22. A significant heterogeneity was found (χ^2^ = 55.88, df = 4, P < 0.001; I^2^ = 93%). (**Figures 5A, B**). The systolic reduction of blood pressure remained significant in a subgroup analysis removing studies with high heterogeneity, MD = 0.10, 95% CI 0.06–17; P <0.001, and Z = 8.38. No significant heterogeneity was found (χ^2^ = 1.64, df = 3, P = 0.65; I^2^ = 0%) (**Figures 5A, B**).

**Figure 6:**
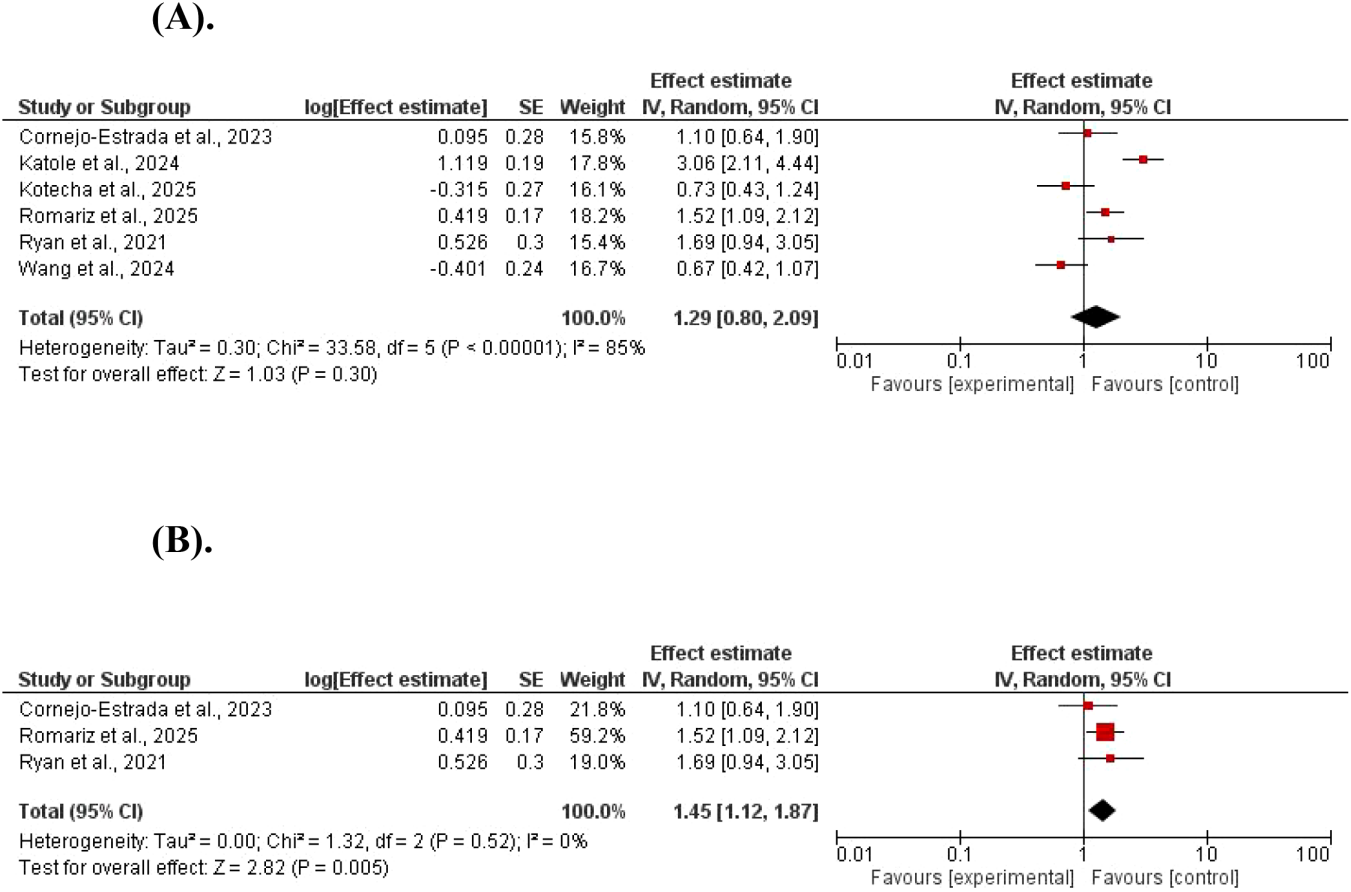
**(A)** The effect of GLP-1 agonists on total adverse events in children. **(B)** The effect of GLP-1 agonists on total adverse events in children (no heterogeneity).

The total adverse events were not different between GLP-1 agonists and controls (log ratios=1.29, 95% CI 0.80–2.09; P = 0.03, and Z = 1.03). A significant heterogeneity was found (χ^2^ = 33.58, df = 5, P < 0.001; I^2^ = 85%). However, the total side effects were higher in GLP-1 agonists after removing studies with high heterogeneity (log ratios=1.45, 95% CI 1.12–1.87; P = 0.005, and Z = 2.82). No significant heterogeneity was found (χ^2^ = 1.32, df = 2, P = 0.52; I^2^ = 0%) (**Figures 6A, B**).

**Figure 7:**
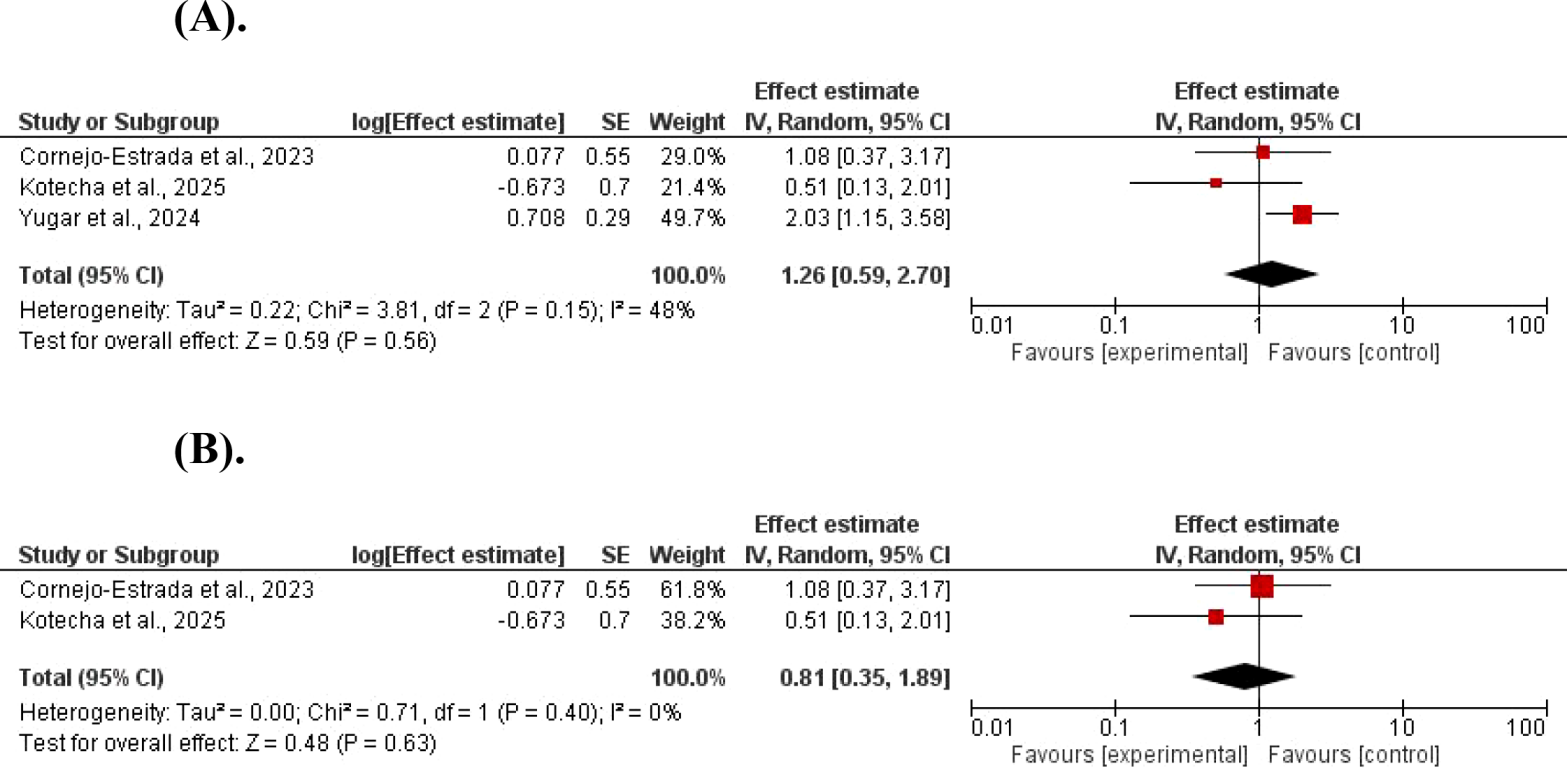
**(A)** The effect of GLP-1 agonists on hypoglycemia in children. **(B)** The effect of GLP-1 agonists on hypoglycemia in children.

Hypoglycemia was not increased in GLP-1 agonists compared to controls (log ratios=1.26, 95% CI 0.59–2.70; P = 0.56, and Z = 0.59). No significant heterogeneity was found (χ^2^ = 3.81, df = 2, P = 0.15; I^2^ = 48%). The results remained insignificant after the elimination of the study with high contribution to heterogeneity (log ratios=0.81, 95% CI 0.35–1.89; P = 0.63, and Z = 0.48). No significant heterogeneity was found (χ^2^ = 0.71, df = 1, P = 0.63; I^2^ = 48%) (**Figures 7A, B**).

## Discussion

In this umbrella review, GLP-1 agonists were more effective than control in reducing body weight, HbA1c, BMI z, and SBP, MD = 0.11, 95% CI 0.05–0.25, MD = 0.65, 95% CI 0.53–0.80, MD = 0.85, 95% CI 0.81–0.90, and MD = 0.19, 95% CI 0.04–83, respectively. The total adverse events and hypoglycemia were not different, log ratios=1.29, 95% CI 0.80–2.09, and log ratios=1.26, 95% CI 0.59–2.70, respectively.

### Evidence from systematic reviews and meta-analyses

Previous meta-analyses have demonstrated significant reductions in body weight (23–27, 29–33, 36). However, findings related to glycemic control remain inconsistent across studies. Moreover, several meta-analyses have not comprehensively evaluated other clinically relevant outcomes, including BMI z score, SBP, total adverse events, and hypoglycemia—parameters that are critical for the optimal management of pediatric obesity and type 2 DM. Consequently, an umbrella review synthesizing all available evidence across these outcomes is both timely and highly warranted.

Regarding the comparative effectiveness of GLP-1 agonists, semaglutide currently has the strongest evidence supporting its efficacy in weight reduction, whereas liraglutide has demonstrated more modest effects, and data on tirzepatide remain preliminary (37). In the context of type 2 DM, liraglutide is the most well-established agent, evidence for tirzepatide is steadily emerging, and data on semaglutide are still limited (38).

Obesity is a chronic inflammatory disease and a biological disease of energy regulation. The pathophysiology is complex and includes genetic, societal, and neuroendocrine interplay. Therefore, the use of GLP-1 agonists is acknowledged because they act in the gastrointestinal tract (39, 40). The use of GLP-1 agonists in children with obesity and type 2 DM is justifiable due to the current evidence.

There is a marked and continuing rise in childhood and adolescent obesity. Currently, hundreds of millions worldwide are either overweight or obese. The prevalence jumped from 8% in 1990 to 20% in 2022, together with parallel increases in youth-onset type 2 DM (8, 41, 42). This trend creates a strong clinical and public-health rationale for considering evidence-based pharmacologic options such as GLP-1 agonists as adjuncts to lifestyle and prevention strategies.

Although lifestyle interventions can result in modest weight reduction, their long-term sustainability remains limited at both individual and population levels. By contrast, randomized controlled trials in adolescents have consistently shown that GLP-1 agonists achieve substantially greater and clinically meaningful reductions in BMI, accompanied by significant improvements in cardiometabolic risk markers, compared with placebo combined with lifestyle modification. The adverse effects associated with GLP-1 agonists are generally acceptable and predominantly gastrointestinal in nature. Accordingly, GLP-1 agonists may be considered for selected adolescents and young individuals with severe obesity or obesity accompanied by comorbidities, with the potential to reduce short-term cardiometabolic risk and to prevent or delay progression to type 2 DM and its related complications (21, 43). The rapidly rising incidence and poorer prognosis of youth-onset Type 2DM mean therapies that safely improve weight and glycemic risk factors could alter lifetime risk trajectories when used under specialist supervision alongside comprehensive care (21, 43, 44).

Evidence from this umbrella review confirmed the benefits of GLP-1 agonists in obesity and diabetes in the short term. In addition, GLP-1 agonists showed benefits in MBMI z and SBP. Moreover, GLP-1 agonists were tolerable, and the total adverse events and hypoglycemia were similar to those of the control. However, there is an increasing concern about medium and long-term safety data, including the effects on growth, pubertal timing, and bone outcomes (12, 45). Importantly, most randomized trials on GLP-1 agonists used in children have a short duration (56–68 weeks), and therefore, they could not assess the effects of puberty timing, final adult height, and bone outcomes that require long-term data. Hypoglycemia with GLP-1 agonist use is rare and mild (5% and 15-20% when combined with insulin and sulphonylurea); no severe hypoglycemia requiring assistance was reported (11, 46). Another major issue is the rising concerns about the association of GLP-1 agonists, depression, and suicidal ideation. Evidence from randomized trials and observational studies showed no association and a possible reduction in the short term. However, long-term results are lacking (47). Potential nutritional deficiencies and effects on peak bone mass are biologically plausible (reduced intake, GI losses, reduced mechanical loading), so active monitoring of growth, diet, and bone-health risk factors is recommended (48). Long-term studies (years to final adult height and bone mass) are needed (49, 50).

Overall, the glycemic efficacy of GLP-1 agonists in children and adolescents with type 2 DM is well established. Consequently, GLP-1 agonists represent a valuable adjunctive therapy for youth with Type 2 DM who exhibit suboptimal glycemic control with metformin or insulin alone. GLP-1agonists (liraglutide, semaglutide, exenatide) are effective adjuncts for pediatric obesity and Type 2 DM, improving weight, glycemic control, and BMI z with modest SBP lowering. Long-term safety regarding growth, puberty, and bone development requires further study. Use should remain individualized and integrated with behavioral therapy, nutrition, and physical activity programs.

The strength of this umbrella review is that it is the first to combine the evidence from 11 meta-analyses (5 were high quality, and three moderate quality). We assessed 4 important morbidities in children with obesity/type 2 DM, including body weight, HbA1c, BMI z, and SBP. In addition, we gave an insight into the total side effects and hypoglycemia.

### Limitations

Most pediatric trials last <1 year, and some of the studies (3) showed low quality. The significant overlap (68 out of 78 trials) significantly limited this umbrella review.

### Conclusion

Based on the low certainty evidence, GLP-1 agonists may improve weight, HbA1c, BMI z-score, and SBP in adolescents with obesity and youth with type 2 DM. Short-term safety outcomes appear acceptable, with no consistent signals of increased adverse events or hypoglycemia compared with standard care. However, the certainty of evidence is limited by study heterogeneity, imprecision, and the lack of long-term pediatric safety data. While semaglutide demonstrates potential comparative advantages in weight-related outcomes, confidence in this finding remains restricted. Consequently, additional well-designed, adequately powered, and long-duration trials are essential before drawing definitive conclusions or informing long-term clinical practice.

## Data Availability

The original contributions presented in the study are included in the article/supplementary material. Further inquiries can be directed to the corresponding author.
